# Supplementary material for: Significant Increase in Clostridioides difficile Mortality During the COVID-19 Pandemic: A Nationwide Study
Source: Gastro Hep Adv. 2026 Mar 30;5(6):100942. doi: 10.1016/j.gastha.2026.100942 (PMC13141497; doi:10.1016/j.gastha.2026.100942)
Supplement: Supplementary Table [file mmc1.pdf]

Supplemental Table 1. ICD-10 codes used for analysis

Diagnosis	ICD-10 codes
C. difficile infection	A04.7 enterocolitis due to *C. difficile*<br>B96.8 other specified bacterial agents as the cause of diarrhea<br>R19.58 other fecal abnormalities
C. difficile carrier status (exclusionary)	Z22.1 carrier of other intestinal infectious diseases<br>Z22 carrier of other specified bacterial diseases<br>Z86.19 personal history of other infectious and parasitic diseases<br>Z87.1 personal history of diseases of the digestive system
