# Supplementary material for: Significant Increase in Clostridioides difficile Mortality During the COVID-19 Pandemic: A Nationwide Study
Source: Gastro Hep Adv. 2026 Mar 30;5(6):100942. doi: 10.1016/j.gastha.2026.100942 (PMC13141497; doi:10.1016/j.gastha.2026.100942)
Supplement: Extended PDF [file mmc2.pdf]

Gastro Hep Advances 2026;5:100942

ORIGINAL RESEARCH—CLINICAL

Significant Increase in *Clostridioides difficile* Mortality During the COVID-19 Pandemic: A Nationwide Study

Jonathan Besney,[1] Christina M. Ray,[2] Katherine A. Buhler,[1] Satchel Krawchuk,[3] Humberto Jijon,[1] Cathy Lu,[1] Cynthia H. Seow,[1] Kerri L. Novak,[1] Joelle St-Pierre,[1] Siddharth Singh,[4] Remo Panaccione,[1] Gilaad G. Kaplan,[1,5] and Christopher Ma[1,5]

[1]*Division of Gastroenterology & Hepatology, Department of Medicine, University of Calgary, Calgary, Alberta, Canada;* [2]*Department of Medicine, University of Calgary, Calgary, Alberta, Canada;* [3]*Faculty of Dentistry, University of Toronto, Toronto, Ontario, Canada;* [4]*Division of Gastroenterology, University of California San Diego, La Jolla, California; and* [5]*Department of Community Health Sciences, University of Calgary, Calgary, Alberta, Canada*

Significant Increase in *Clostridioides difficile* Mortality During the COVID-19 Pandemic: A Nationwide Study

Aim: evaluate national patterns in CDI-associated hospitalizations before and during the COVID-19 pandemic

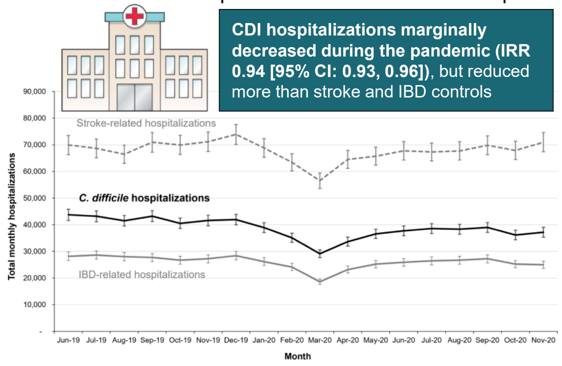

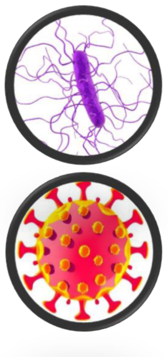

Mortality in CDI-associated hospitalizations:
Pre-pandemic 4.2% vs. pandemic 5.1% (p<0.0001)

Adjusted risk of in-hospital mortality	aOR [95% CI] in patients with CDI	aOR [95% CI] in patients with COVID-19
Concomitant COVID-19	5.33 [4.39, 6.48] p<0.001	-
Concomitant *Clostridioides difficile*	-	1.48 [1.36, 1.60] p<0.001

BACKGROUND AND AIMS: *Clostridioides difficile* infection (CDI) is the most common nosocomial infectious diarrhea and is associated with high morbidity and mortality. There are conflicting data on the impact of Coronavirus Disease 2019 (COVID-19) on CDI. We aimed to evaluate national patterns in CDI-associated hospitalizations before and after the pandemic. **METHODS:** Data from the National Inpatient Sample between January 2018 and December 2020 were analyzed. Temporal trends in admissions before and after the pandemic for CDI were analyzed using survey-adjusted Poisson regression and compared to hospitalizations for patients admitted for stroke and inflammatory bowel disease as controls. Univariable and multivariable logistic regression was used to evaluate mortality in CDI-associated admissions before and after the pandemic, adjusting for COVID-19. A bidirectional relationship was also explored, evaluating mortality in COVID-19-related admissions complicated by CDI. **RESULTS:** Rates of CDI-related hospitalizations decreased marginally during the pandemic (incidence rate ratio 0.94 [95% confidence interval [CI]: 0.93–0.96], $P < .001$); however, this was a significant decrease compared to hospitalization volumes for stroke ($P = .01$) and inflammatory bowel disease ($P = .05$) during initial phase of the pandemic. Mortality during the pandemic for CDI-associated hospitalizations increased (5.1% vs 4.2%); on multivariable logistic regression, this was most significant in patients with concomitant COVID-19 (adjusted odds ratio 5.33 [95% CI: 4.39–6.48], $P < .001$). In patients primarily admitted for COVID-19 who concurrently had CDI, risk of mortality also increased significantly (adjusted odds ratio 1.48 [95% CI: 1.36–1.61], $P < .001$). **CONCLUSION:** Significantly increased risks of mortality were observed in CDI-associated hospitalizations with concomitant COVID-19 and vice versa.

Keywords: Clostridium; *C difficile*; COVID-19; Death; Epidemiology; Inpatient

Abbreviations used in this paper: **ACE2, angiotensin-converting enzyme 2; AMPC, average monthly percent change; aOR, adjusted odds ratio; CDI, *Clostridioides difficile* infection; CI, confidence interval; COVID-19, coronavirus disease 2019; IBD, inflammatory bowel disease; ICD, International Classification of Diseases; IPC, infection prevention and control; IRR, incidence rate ratio; NIS, National Inpatient Sample; OR, odds ratio; SARS-CoV-2, severe acute respiratory syndrome coronavirus 2.**

Most current article

© 2026 The Authors. Published by Elsevier Inc. on behalf of American Gastroenterological Association Institute. This is an open access article under the CC BY license (http://creativecommons.org/licenses/by/4.0/).
2772-5723
https://doi.org/10.1016/j.gastha.2026.100942

Introduction

Clostridioides difficile infection (CDI) is the most common cause of nosocomial infectious diarrhea and leads to substantial burdens on the health-care system with an estimated excess cost of $4.8 billion in the United States annually.[1,2] Horizontal spread by *C difficile* spores may be mitigated by appropriate handwashing and contact precautions.[3] However, despite widespread antimicrobial stewardship efforts and infection prevention and control (IPC) measures, CDI still affects an estimated 3–4 per 10,000 patient days per year and increases the risk of both all-cause and cause-specific mortality by ∼3- to 7-fold.[4–6] Patient groups at especially high risk include the elderly, immunocompromised, medically comorbid, and those requiring antibiotics or prolonged hospitalization.[7–9]

During the beginning of the Coronavirus Disease 2019 (COVID-19) pandemic, there was a systematically heightened focus on IPC measures to reduce the transmission of the severe acute respiratory syndrome coronavirus 2 (SARS-CoV-2) virus, particularly during the first wave of lockdown measures. Previous reports documented that adherence to appropriate hand hygiene improved; however, this diligence was not sustained later into the pandemic.[10] It was hypothesized that more consistent handwashing and IPC adherence might result in reduced rates of nosocomial CDI. However, data were conflicting, with authors reporting both increased and decreased rates of infection in different study populations.[11]

Here, we aimed to evaluate CDI rates nationally, both prior to, during, and after the first wave of the pandemic. We compared the change in rate of CDI infections requiring hospitalization with patients hospitalized for both gastrointestinal and nongastrointestinal indications to assess whether temporal patterns were unique to CDI or reflective of other systematic patterns. Finally, we evaluated factors associated with mortality in patients with CDI in the context of the pandemic, and the relationship between CDI, COVID-19 infection, and mortality.

Methods

Study Design and Data Source

We conducted a retrospective analysis of data collected in the Healthcare Cost and Utilization Project National Inpatient Sample (NIS) between January 2018 and December 2020. The NIS is the largest, publicly available, administrative health database in the United States. Each year, data are collected from more than 7 million hospital admissions from over 1000 community hospitals across the country. Data from the NIS are estimated to cover approximately 98% of the US population. The NIS approximates a 20% stratified sample of discharges; the sampling design allows capture of data on rare conditions and special patient populations, with subsequent weighting applied to generate representative population-based estimates.

Patient Population and Outcomes

Adult patients aged ≥18 years with a diagnosis of CDI were identified using International Classification of Diseases (ICD) 10th Revision Clinical Modification diagnostic codes (Supplementary Table). Recognizing that patients may be admitted with another medical or surgical condition and have their course complicated by CDI, we included admissions with CDI in any diagnostic position; however, we attempted to minimize potential inclusion of patients with CDI carrier status by excluded patients with ICD-10 codes for carrier status or personal history but inactive infectious or bacterial diseases. Previous studies have demonstrated that ICD coding is moderately sensitive and highly specific (>95%) for CDI; accordingly, our estimates of incidence are likely to be underestimated.[12,13]

To evaluate whether temporal trends in CDI-associated hospitalizations were driven by more generalized effects on health systems due to the pandemic, we a priori evaluated the same patterns in 2 control populations: those admitted with a primary diagnosis of stroke and those admitted with a primary diagnosis of inflammatory bowel disease (IBD) (Crohn's disease or ulcerative colitis). Stroke was identified as a comparator group because rates of admission rates during the pandemic may reflect system-related trends observed during this time (eg, increased patient hesitancy to seek out healthcare) but would be unrelated to better hygiene-based practices or antibiotic prescribing (the primary risk factors for CDI).[14,15] IBD was chosen as a second comparator group as this is a predominantly gastrointestinal condition where management may have been preferentially directed to outpatient care during the pandemic.

Statistical Analysis

Survey weights provided by the Health Care Utilization Project were applied to all analyses to generate population-representative estimates. Descriptive statistics were used to summarize patient characteristics and comparisons in baseline demographics were performed using analyses adjusted for the survey design. Temporal trends in CDI-associated hospitalizations were expressed as an average monthly percent change (AMPC), calculated using a generalized linear model assuming a Poisson distribution. We evaluated hospitalization patterns before and after 2020, recognizing that there was a substantive disruption to health-care service delivery during the pandemic. Differences in hospitalization rates were expressed as an incidence rate ratio (IRR) with 95% confidence intervals (CI), and IRRs for CDI-associated hospitalizations were compared against those for stroke and IBD to determine if patterns during the pandemic were specific to CDI or related to system-level observations.

The relationship between CDI, COVID-19 infection, and care delivery during the pandemic on all-cause mortality in CDI-associated hospitalizations was then evaluated in univariable and multivariable survey-weighted logistic regression. Both patient and system-related covariables were included in modeling. We hypothesized that there may be bidirectional relationships between the risk of mortality in patients with CDI and COVID-19. Therefore, we assessed both the adjusted risk of mortality in patients with CDI and concurrent COVID-19, as well as the risk of mortality in patients admitted for a primary diagnosis of COVID-19 who also had CDI. Finally, we conducted a sensitivity analysis to evaluate the impact of geographic region on mortality, recognizing that the response to COVID-19 varied in

Table 1. Demographic Characteristics Associated With Hospital Admissions for *Clostridioides difficile*, Before and After Onset of the COVID-19 Pandemic

Characteristic	Prepandemic (January 2018 to January 2020)	Postpandemic onset (February to December 2020)	*P* value
Total admissions for CDI			–
Unweighted observations (n)	219,961	80,340	
Weighted observations (N)	1,099,805	401,700	
Mean age (y, 95% CI)	64.6 [64.4–64.7]	64.6 [64.4–64.9]	.94
Female sex (%, 95% CI)	54.7 [54.4–54.9]	53.0 [52.6–53.3]	<.001
Race (%, 95% CI)			.60
White	69.3 [68.6–70.1]	68.3 [67.2–69.4]	
Black	15.3 [14.8–15.9]	15.7 [14.9–16.5]	
Hispanic	9.8 [9.3–10.2]	10.1 [9.4–10.8]	
Asian or Pacific Islander	2.2 [2.0–2.4]	2.3 [2.1–2.6]	
Other	2.6 [2.3–2.8]	2.7 [2.4–3.0]	
Elixhauser Comorbidity Index (%, 95% CI)			<.001
0	3.2 [3.1–3.3]	2.7 [2.6–2.9]	
1	6.7 [6.6–6.8]	5.9 [5.7–6.1]	
≥2	90.1 [90.0–92.3]	91.4 [91.1–91.6]	
Comorbid IBD (%, 95% CI)	3.1 [3.0–3.1]	3.0 [2.9–3.2]	.91
Primary payment method			<.001
Medicare	61.7 [61.3–62.1]	60.1 [59.5–60.8]	
Medicaid	14.4 [14.1–14.7]	15.3 [14.8–15.8]	
Private insurance	18.2 [17.8–18.6]	18.5 [18.0–19.0]	
Other	2.2 [2.1–2.3]	2.6 [2.4–2.8]	
Median household income by ZIP code			.34
0–25th percentile	30.9 [30.1–31.6]	30.8 [29.7–32.0]	
26–50th percentile	26.7 [26.2–27.3]	27.8 [26.9–28.6]	
51–75th percentile	23.6 [23.1–24.0]	22.9 [22.1–23.6]	
75–100th percentile	18.9 [18.2–19.5]	18.6 [17.5–19.7]	
Hospital bed size			.39
Small	21.1 [20.3–21.8]	22.3 [20.9–23.8]	
Medium	28.3 [27.4–29.3]	27.1 [25.4–28.9]	
Large	50.6 [49.5–51.8]	50.5 [48.3–52.6]	
Teaching hospital status			.36
Rural	9.8 [9.3–10.3]	9.7 [8.9–10.6]	
Urban nonteaching	19.1 [18.4–19.9]	17.9 [16.6–19.4]	
Urban teaching	71.1 [70.2–72.0]	72.4 [70.7–74.0]	
Hospital region			.99
Northeast	18.0 [17.1–18.9]	18.0 [16.4–19.7]	
Midwest	24.3 [23.3–25.4]	24.1 [22.3–26.0]	
South	39.1 [38.0–40.3]	38.9 [36.8–41.0]	
West	18.6 [17.8–19.4]	19.0 [17.4–20.7]	

different parts of the country. Here, we evaluated for effect modification between region (defined by the U.S. Census Bureau as Northeast, Midwest, South, and West) and COVID-19 infection in CDI-associated hospitalizations. Significant effect modification was determined based on testing for significance ($P < .05$) of the interaction term using the Wald test.

All analyses were performed using STATA 17.0 (StataCorp LLC, College Station, TX) and Joinpoint Regression Program 4.6 (Statistical Research and Applications Branch, National Cancer Institute).

Ethical Considerations

Institutional review board ethics approval was not required for this study using deidentified, admission-based data.

Results

Demographic Characteristics

A total of 300,301 unweighted hospital admissions for CDI, representing an estimated ∼1,501,505 weighted admissions during the study period were assessed. Demographic characteristics are summarized in Table 1. The mean age at discharge was 64.6 years [95% CI: 64.4–64.8] and 54.2% [95% CI: 54.0%–54.4%] of patients were female. Most admissions involved patients who were significantly comorbid (90.4% [95% CI: 90.3%–90.6%] with Elixhauser comorbidity index ≥2) and approximately 3% of admissions for CDI involved patients with comorbid IBD. A total of 5.0% [95% CI: 4.8%–5.2%] of admissions associated with CDI were complicated by COVID-19 infection. Patients primarily were managed in larger hospitals, with

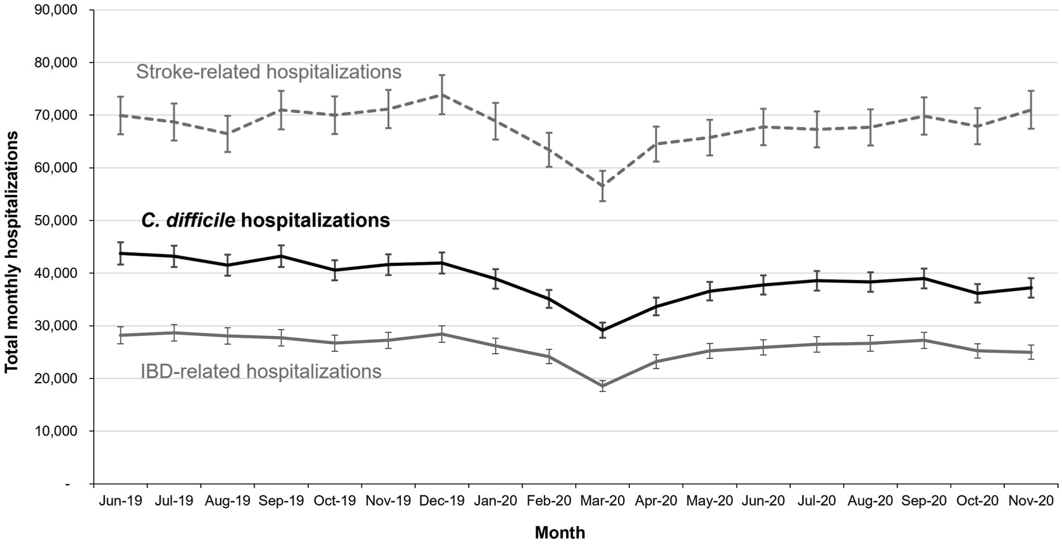

Figure. Monthly hospitalizations for CDI, IBD, and stroke from June 2019 through November 2020. IBD, inflammatory bowel disease.

71.4% [95% CI: 70.8%–72.0%] of cases being managed in urban teaching hospitals.

Trends in Clostridioides difficile *Infection Incidence*

Prepandemic rates of CDI-associated hospitalization were slowly decreasing (AMPC −0.5% [95% CI: −0.3% to −0.8%], $P < .001$) as compared to the slowly increasing rates of IBD- and stroke-related hospitalizations (AMPC +0.3% [95% CI: +0.1% to +0.5%], $P = .01$ for both conditions). In 2020, there was a substantial disruption in the pattern of hospitalization volumes. Figure demonstrates an initial reduction in hospitalization volume across indications but subsequently stabilized rates of hospitalization for CDI (AMPC −0.1% [95% CI: −1.7% to +1.5%], $P = .88$), IBD (AMPC −0.9% [95% CI: −4.5% to +2.7%, $P = .61$), and stroke (AMPC 0.0% [95% CI: −0.1% to +0.2%], $P = .77$) in 2020. Compared to prepandemic, hospitalization incidence rates for CDI (IRR 0.94 [95% CI: 0.93–0.96], $P < .001$), IBD (IRR 0.97 [95% CI: 0.94–1.00], $P = .03$), and stroke (IRR 0.97 [95% CI: 0.95–0.99], $P = .01$) all decreased during 2020. The reduction in CDI-associated hospitalization volume was significantly greater than that observed for both IBD ($P = .05$) and for stroke ($P = .01$), although the absolute magnitude of this effect was small.

Mortality Risks in Clostridioides difficile *Infection–Associated Hospitalizations*

Approximately 4.5% [95% CI: 4.3%–4.6%] of admissions with CDI resulted in in-hospital mortality. This increased significantly from before the pandemic (4.2%) to during the pandemic (5.1%) ($P < .0001$), and on univariable logistic regression, admission during 2020 increased the unadjusted odds of mortality by 23% (odds ratio [OR] 1.23 [95% CI: 1.17–1.30], $P < .001$). However, this observation was predominantly driven by the impact of COVID-19. In univariable analysis, COVID-19 was associated with an increased risk of mortality (OR 5.91 [95% CI: 5.45–6.40], $P < .001$). After adjusting for patient- and system-level confounders in multivariable analysis, time of admission (pre- vs postonset of the pandemic) was not associated with mortality (adjusted OR [aOR] 0.98 [95% CI: 0.94–1.03], $P = .54$) (Table 2). However, concurrent COVID-19 continued to increase the risk of mortality in hospitalizations associated with CDI by over 5-fold (aOR 5.33 [95% CI: 4.39–6.48], $P < .001$).

COVID-19 was the dominant factor associated with mortality in CDI-related hospitalizations, and the magnitude of this effect was more significant than all other predictors of mortality, including older age, male sex, non-White race, presence of comorbidities, non-Medicare payment, lower socioeconomic status by estimated median household income by ZIP code, and hospital volume (Table 2). In a sensitivity analysis, we evaluated for potential effect modification between COVID-19 and geographic region on mortality risk in CDI-related hospitalizations: there was no significant effect modification and across all regions, COVID-19 significantly increased the risk of death by 5- to 6-fold (aOR in the Northeast region 5.26 [95% CI: 4.30–6.44], $P < .001$; Midwest region 6.11 [95% CI: 5.18–7.23], $P < .001$; South region 5.68 [95% CI: 4.91–6.56], $P < .001$; West region 5.47 [95% CI: 4.44–6.76], $P < .001$).

Impact of Clostridioides difficile *Infection on COVID-19-Related Mortality*

We also examined the bidirectional relationship of CDI on hospitalizations for COVID-19. Amongst admissions with a primary diagnosis of COVID-19, mortality rates were

Table 2. Factors Associated With In-Hospital Mortality in Admissions With CDI and COVID-19 in Multivariable Analysis

Covariable	Multivariable aOR for CDI-associated hospitalizations [95% CI]	*P* value	Multivariable aOR for COVID-19 hospitalizations [95% CI]	*P* value
Age (per decade)	1.29 [1.27–1.31]	<.001	1.58 [1.56–1.60]	<.001
Female sex	0.86 [0.83–0.89]	<.001	0.70 [0.69–0.72]	<.001
Race				
White	Reference	–	Reference	–
Black	1.09 [1.03–1.15]	<.01	1.08 [1.04–1.12]	<.001
Hispanic	1.01 [0.95–1.09]	.68	1.32 [1.27–1.38]	<.001
Asian or Pacific Islander	1.25 [1.12–1.40]	<.001	1.22 [1.14–1.31]	<.001
Other	1.21 [1.09–1.36]	<.001	1.31 [1.23–1.40]	<.001
Elixhauser Comorbidity Index				
0	Reference	–	Reference	–
1	1.80 [1.25–2.60]	<.01	1.62 [1.54–1.71]	<.001
≥2	8.08 [5.77–11.32]	<.001	2.08 [1.84–2.35]	<.001
Inflammatory bowel disease	0.87 [0.76–0.99]	.03	1.00 [0.86–1.15]	.97
Primary payment method				
Medicare	Reference	–	Reference	–
Medicaid	1.12 [1.04–1.20]	<.01	1.13 [1.08–1.19]	<.001
Private insurance	1.13 [1.07–1.20]	<.001	0.99 [0.94–1.03]	.56
Other	1.82 [1.63–2.05]	<.001	1.62 [1.49–1.75]	<.001
Median household income by ZIP code				
0–25th percentile	Reference	–	Reference	–
26–50th percentile	0.91 [0.86–0.96]	<.001	0.89 [0.85–0.92]	<.001
51–75th percentile	0.90 [0.85–0.95]	<.001	0.81 [0.78–0.85]	<.001
75–100th percentile	0.89 [0.85–0.95]	<.001	0.77 [0.73–0.81]	<.001
Hospital bed size				
Small	Reference	–	Reference	–
Medium	1.24 [1.17–1.32]	<.001	1.17 [1.11–1.23]	<.001
Large	1.43 [1.35–1.51]	<.001	1.20 [1.14–1.26]	<.001
Teaching hospital status				
Rural	Reference	–	Reference	–
Urban nonteaching	1.38 [1.27–1.52]	<.001	1.34 [1.24–1.44]	<.001
Urban teaching	1.73 [1.60–1.88]	<.001	1.52 [1.43–1.62]	<.001
Pre- vs postpandemic	0.98 [0.94–1.03]	.54	–	–
COVID-19 infection	5.33 [4.39–6.48]	<.001	–	–
Clostridioides difficile infection	–	–	1.48 [1.36–1.60]	<.001

significantly higher in patients who concurrently had CDI (20.9% vs 11.1%, $P < .0001$). Similar results were observed in admissions with any diagnostic code for COVID-19 (20.6% vs 13.3%, $P < .0001$). In multivariable logistic regression, CDI significantly increased the risk of mortality in admissions for COVID-19 (aOR 1.48 [95% CI: 1.36–1.61], $P < .001$). The magnitude of the effect of CDI on mortality in COVID-19–related admissions was similar to that observed for older age (aOR 1.58 per decade [95% CI: 1.56–1.60], $P < .001$) and multicomorbidity (aOR 2.08 [95% CI: 1.84–2.35], $P < .001$).

Discussion

The COVID-19 pandemic had extensive and systematic effects on public health measures and the burden experienced by health systems. Conditions during the pandemic, including both patient- and system-related factors, were likely to impact the epidemiology of CDI. In this analysis of over 1.5 million admissions for CDI both before and during the first year of the pandemic, we evaluated temporal patterns in CDI-associated hospitalizations and the risk of mortality. We showed that prior to the pandemic, rates of CDI were slowly decreasing; however, there was a significant disruption to the pattern of hospital admissions in 2020. Overall, there was a reduction in total CDI-associated hospitalization volume, both compared to prepandemic and compared to admissions for stroke and IBD, although the absolute magnitude of this effect was small (~6%). Strikingly, mortality during the pandemic for CDI-associated hospitalizations significantly increased: on multivariable regression, this effect was dominated by the impact of having concomitant COVID-19 infection. However, our findings do not simply reflect higher mortality because of COVID-19. Indeed, this relationship was bidirectional, with patients admitted primarily for COVID-19 who had concomitant CDI also having increased rates of in-hospital death. Overall, our findings provide clarity on a national level for the impact of COVID-19 on CDI-associated hospitalizations and their outcomes.

Several studies have previously examined rates of CDI during the COVID-19 pandemic, with conflicting results. For example, McMullen et al[16] first reported 45%–51% reductions very early in the pandemic (published in July 2020), and Weiner-Lastinger et al reported reductions in laboratory confirmed CDI of −12.1% to −14.2% in the first 2 quarters of 2020, despite increased risks of central-line associated bloodstream infections, catheter-associated urinary tract infections, and ventilator-associated events.[17] In contrast, other authors have described significantly increased or unchanged rates of CDI incidence or increased complications related to CDI in the context of COVID-19 infection.[18–22] Granata et al[23] synthesized data from 13 retrospective cohort studies, capturing the time period between February 2020 and February 2021, reporting pooled incidence rates ranging from 1.4 to 4.4 CDI cases per 10,000 patient days, which was largely unchanged compared to prepandemic. However, most of these studies were single center or captured regional experiences, which may not be representative of other jurisdictions.

Our findings show that on a national level in the United States, a statistically lower rate of CDI-associated hospitalizations was observed during 2020, both compared to before the pandemic and when compared to hospitalizations for patients with IBD and stroke. However, the absolute magnitude of this reduction was small (IRR 0.94), and temporally, although there was a disruption in hospitalization pattern in 2020, total hospitalization volumes returned to stable, near-prepandemic levels by mid-2020. We hypothesize that multiple factors are likely contributing to heterogeneity in the literature and to our observed trends. Early in the pandemic, fewer patients were presenting to hospital due to concerns around contracting COVID-19, or they were being managed in the community with both patients and providers looking to avoid hospitalization.[24] Overall hospitalization volumes declined in the first half of 2020 across all demographic subgroups by >20%, even among patients with acute medical conditions, as health systems diverted resources toward management of respiratory and critical care illnesses.[25] Reductions in CDI testing have previously been reported due to attribution of gastrointestinal symptoms to COVID-19 infection rather than to gastrointestinal pathology.[26,27] Finally, social distancing and lockdown effects and improved adherence to IPC measures may have helped decrease horizontal transmission of CDI early in the pandemic, although these measures were not sustained for subsequent waves of COVID-19.

Contrastingly, other factors likely offset any reductions in CDI incidence during the pandemic. Despite the lack of evidence, many patients with COVID-19 were treated with broad spectrum antimicrobials for presumed superimposed bacterial infections.[28] Granata et al[20] hypothesized that hospitalization itself, with alterations in the gut microbiome from the use of broad-spectrum antimicrobials contributed to the development of nosocomial CDI. While it is well established that CDI requires the use of proper handwashing and personal protective equipment to prevent spread, Yadlapati et al[26] suggested that alcohol-based hand hygiene, which was primarily used to prevent the spread of COVID-19, was insufficient to decrease transmission of *C difficile* spores and rates of compliance with hand hygiene decreased later in the pandemic.[29]

An important observation in our analysis is that mortality in CDI-associated hospitalizations increased after the onset of the COVID-19 pandemic, even after adjusting for important potential confounders such as age, comorbidity, and hospital setting.[30] In multivariable analysis, we show that the impact on mortality in CDI-associated hospitalizations was driven by a >5-fold increased risk of death in patients with concomitant COVID-19, and that this relationship was bidirectional, with a 50% increased risk of mortality in patients admitted for COVID-19 who develop CDI. These findings are consistent with an analysis of over 8 million Medicare admissions to US hospitals, evaluating pre- and postpandemic mortality from non-SARS-CoV-2 causes by Dang et al.[31] Compared to 2019, admissions for almost all evaluated diagnoses, ranging from chest pain to gastrointestinal bleeding, increased by approximately 10%–30%, and this effect was most pronounced amongst racial minorities, those with low socioeconomic status, and in lower-quality hospitals or those with high caseloads of SARS-CoV-2.[31] We hypothesize that our observation that COVID-19 worsens outcomes in CDI-associated hospitalizations (and vice versa) relate to the disproportionate impact of both diseases on older and medically frail patients, increased risk of antibiotic exposures, and potential preferential admission of sicker patients with more severe CDI to hospital during the pandemic.[32,33]

We also considered other factors that may have played a role in the observed higher rates of mortality, although were not evaluable directly in this dataset. For example, there may have been limited operating room availability during the pandemic, reducing access to colectomy for CDI-related complications such as toxic megacolon.[34] Changes in physical examination practices during the pandemic may have led to delayed detection of surgical indications in patients with severe CDI.[35] Patients with CDI and COVID-19 coinfection may have been poor surgical candidates resulting from their respiratory status or other comorbidities. Access to fecal microbial transplant as a therapeutic option for CDI may have been reduced during the pandemic.[36] Finally, there is a biological interaction between COVID-19 and the gastrointestinal tract, which impacts transmissibility, symptoms, and alterations of the fecal microbiome. Mechanistically, the SARS-CoV-2 virus enters the enterocyte via the angiotensin-converting enzyme 2 (ACE2) which is highly expressed in the gastrointestinal tract, allowing for viral replication.[37,38] The literature suggests that gut microbiota may affect ACE2 expression, which may have been impacted either by CDI, antibiotic use, altered hygiene, and in some cases hospitalization. Additionally, it is possible dysbiosis resulting from COVID-19 infection may predispose to CDI. It is

possible that these changes in the gut microbiome, leading to altered ACE2 receptor expression may have affected the risk, severity, and mortality from CDI.[39]

Our study has several strengths. We evaluated nationally representative data, collected before, during, and after the first wave of the COVID-19 pandemic, and compared rates of CDI-associated hospitalizations with controls to evaluate disease- and system-related trends in outcomes. However, we acknowledge several important limitations. Our analysis is based on visit-level data, rather than individual patient-level analysis. Therefore, we are unable to adjust for more granular potential confounders such as antibiotic exposure. Additionally, as all data in NIS are deidentified and collected at the level of the hospitalization, we are unable to account for recurrent hospitalizations for a single patient, which is relevant given the increased risk of refractory and recurrent CDI. Third, we evaluated all hospital admissions associated with CDI; however, the lack of a "present-on-admission flag" in NIS data precludes us from evaluating which patients are admitted specifically for CDI as compared to patients with comorbid CDI or those with complications from CDI developing during admission. However, we were careful to exclude patients with diagnostic coding for *C difficile* carrier status. Finally, all administrative data studies are subject to potential errors in misclassification although prior validation studies have demonstrated reasonable sensitivity and high specificity for CDI using ICD-10 coding.

Conclusion

In conclusion, rates of CDI admissions marginally decreased during the pandemic nationally. However, the risk of mortality in hospitalizations associated with CDI increased significantly after the pandemic, driven predominantly by patients coinfected with SARS-CoV-2. The reciprocal finding was also true, with increased mortality observed in patients primarily admitted for COVID-19 who have concurrent CDI. Our findings emphasize the importance of ongoing efforts toward early recognition, diagnosis, and appropriate treatment of CDI, especially in patients with COVID-19. Furthermore, attention to managing coinfections will be critical to improving outcomes in potential future pandemics.

Supplementary Materials

Material associated with this article can be found, in the online version, at https://doi.org/10.1016/j.gastha.2026.100942.

References

1. Dubberke ER, Olsen MA. Burden of Clostridium difficile on the healthcare system. Clin Infect Dis 2012;55(Suppl 2):S88–S92.
2. Smits WK, Lyras D, Lacy DB, et al. Clostridium difficile infection. Nat Rev Dis Primers 2016;2:16020.
3. Kiersnowska ZM, Lemiech-Mirowska E, Michalkiewicz M, et al. Hand hygiene as the basic method of reducing Clostridium difficile infections (CDI) in a hospital environment. Ann Agric Environ Med 2021;28:535–540.
4. Balsells E, Shi T, Leese C, et al. Global burden of Clostridium difficile infections: a systematic review and meta-analysis. J Glob Health 2019;9:010407.
5. Jen MH, Saxena S, Bottle A, et al. Increased health burden associated with Clostridium difficile diarrhoea in patients with inflammatory bowel disease. Aliment Pharmacol Ther 2011;33:1322–1331.
6. Boven A, Vlieghe E, Engstrand L, et al. Clostridioides difficile infection-associated cause-specific and all-cause mortality: a population-based cohort study. Clin Microbiol Infect 2023;29:1424–1430.
7. Eze P, Balsells E, Kyaw MH, et al. Risk factors for Clostridium difficile infections - an overview of the evidence base and challenges in data synthesis. J Glob Health 2017;7:010417.
8. Balram B, Battat R, Al-Khoury A, et al. Risk factors associated with Clostridium difficile infection in inflammatory bowel disease: a systematic review and meta-analysis. J Crohns Colitis 2019;13:27–38.
9. Rodriguez C, Romero E, Garrido-Sanchez L, et al. Microbiota insights in Clostridium difficile infection and inflammatory bowel disease. Gut Microbes 2020;12: 1725220.
10. Moore LD, Robbins G, Quinn J, et al. The impact of COVID-19 pandemic on hand hygiene performance in hospitals. Am J Infect Control 2021;49:30–33.
11. Spigaglia P. Clostridioides difficile infection (CDI) during the COVID-19 pandemic. Anaerobe 2022;74:102518.
12. Dubberke ER, Reske KA, McDonald LC, et al. ICD-9 codes and surveillance for Clostridium difficile-associated disease. Emerg Infect Dis 2006;12:1576–1579.
13. Pfister T, Rennert-May E, Ellison J, et al. Clostridioides difficile infections in Alberta: the validity of administrative data using ICD-10 diagnostic codes for CDI surveillance versus clinical infection surveillance. Am J Infect Control 2020;48:1431–1436.
14. Van Dusen RA, Abernethy K, Chaudhary N, et al. Association of the COVID-19 pandemic on stroke admissions and treatment globally: a systematic review. BMJ Open 2023;13:e062734.
15. Kristoffersen ES, Jahr SH, Faiz KW, et al. Stroke admission rates before, during and after the first phase of the COVID-19 pandemic. Neurol Sci 2021; 42:791–798.
16. McMullen KM, Smith BA, Rebmann T. Impact of SARS-CoV-2 on hospital acquired infection rates in the United States: predictions and early results. Am J Infect Control 2020;48:1409–1411.
17. Weiner-Lastinger LM, Pattabiraman V, Konnor RY, et al. The impact of coronavirus disease 2019 (COVID-19) on healthcare-associated infections in 2020: a summary of data reported to the National Healthcare Safety Network. Infect Control Hosp Epidemiol 2022; 43:12–25.
18. Tariq R, Saha S, Furqan F, et al. Prevalence and mortality of COVID-19 patients with gastrointestinal

symptoms: a systematic review and meta-analysis. Mayo Clin Proc 2020;95:1632–1648.
19. Paramo-Zunzunegui J, Ortega-Fernandez I, Calvo-Espino P, et al. Severe Clostridium difficile colitis as potential late complication associated with COVID-19. Ann R Coll Surg Engl 2020;102:e176–e179.
20. Granata G, Bartoloni A, Codeluppi M, et al. The burden of Clostridioides difficile infection during the COVID-19 pandemic: a retrospective case-control study in Italian hospitals (CloVid). J Clin Med 2020;9:3855.
21. Baccolini V, Migliara G, Isonne C, et al. The impact of the COVID-19 pandemic on healthcare-associated infections in intensive care unit patients: a retrospective cohort study. Antimicrob Resist Infect Control 2021;10:87.
22. Zouridis S, Sangha M, Feustel P, et al. Clostridium difficile infection rates during the pandemic in New York capital area: a single-center study. Cureus 2023;15:e37576.
23. Granata G, Petrosillo N, Al Moghazi S, et al. The burden of Clostridioides difficile infection in COVID-19 patients: a systematic review and meta-analysis. Anaerobe 2022;74:102484.
24. Nourazari S, Davis SR, Granovsky R, et al. Decreased hospital admissions through emergency departments during the COVID-19 pandemic. Am J Emerg Med 2021;42:203–210.
25. Birkmeyer JD, Barnato A, Birkmeyer N, et al. The impact of the COVID-19 pandemic on Hospital admissions in the United States. Health Aff (Millwood) 2020;39:2010–2017.
26. Yadlapati S, Jarrett SA, Lo KB, et al. Examining the rate of Clostridioides (formerly Clostridium) difficile infection pre- and post-COVID-19 pandemic: an institutional review. Cureus 2021;13:e20397.
27. Allegretti JR, Nije C, McClure E, et al. Prevalence and impact of Clostridioides difficile infection among hospitalized patients with coranavirus disease 2019. JGH Open 2021;5:622–625.
28. Beovic B, Dousak M, Ferreira-Coimbra J, et al. Antibiotic use in patients with COVID-19: a 'snapshot' Infectious Diseases International Research Initiative (ID-IRI) survey. J Antimicrob Chemother 2020;75:3386–3390.
29. Makhni S, Umscheid CA, Soo J, et al. Hand hygiene compliance rate during the COVID-19 pandemic. JAMA Intern Med 2021;181:1006–1008.
30. Abou Chakra CN, Pepin J, Sirard S, et al. Risk factors for recurrence, complications and mortality in Clostridium difficile infection: a systematic review. PLoS One 2014;9:e98400.
31. Dang A, Thakker R, Li S, et al. Hospitalizations and mortality from non-SARS-CoV-2 causes among Medicare beneficiaries at US hospitals during the SARS-CoV-2 pandemic. JAMA Netw Open 2022;5:e221754.
32. Janke AT, Mei H, Rothenberg C, et al. Analysis of hospital resource availability and COVID-19 mortality across the United States. J Hosp Med 2021;16:211–214.
33. Reveles KR, Frei AL, Strey KA, et al. Prevalence and health outcomes of Clostridioides difficile infection during the coronavirus disease 2019 pandemic in a national sample of United States hospital systems. Open Forum Infect Dis 2022;9:ofac441.
34. Purdy AC, Smith BR, Hohmann SF, et al. The impact of the novel coronavirus pandemic on gastrointestinal operative volume in the United States. Surg Endosc 2022;36:1943–1949.
35. Erdevir M, Uyaroglu OA, Ozdede M, et al. "COVID-19: the final nail in the coffin for physical examination" Evaluation of the effects of COVID-19 pandemic on physical examination habits of residents in a university hospital: a cross-sectional survey. Int J Clin Pract 2021;75:e14988.
36. Groenewegen B, van Lingen E, Ooijevaar RE, et al. How to prepare stool banks for an appropriate response to the ongoing COVID-19 pandemic: experiences in the Netherlands and a retrospective comparative cohort study for faecal microbiota transplantation. PLoS One 2022;17:e0265426.
37. Xu J, Chu M, Zhong F, et al. Digestive symptoms of COVID-19 and expression of ACE2 in digestive tract organs. Cell Death Discov 2020;6:76.
38. Edwinson A, Yang L, Chen J, et al. Colonic expression of Ace2, the SARS-CoV-2 entry receptor, is suppressed by commensal human microbiota. Gut Microbes 2021;13:1984105.
39. Zhang F, Lau RI, Liu Q, et al. Gut microbiota in COVID-19: key microbial changes, potential mechanisms and clinical applications. Nat Rev Gastroenterol Hepatol 2023;20:323–337.

Received November 8, 2025. Accepted March 20, 2026.

Correspondence:
Address correspondence to: Christopher Ma, MD, MPH, Departments of Medicine and Community Health Sciences, University of Calgary, 6D21 CWPH Building, 3280 Hospital Drive NW, Calgary, Alberta T2N 4Z6, Canada. e-mail: christopher.ma@ucalgary.ca.

Authors' Contributions:
Jonathan Besney: Data collection, data analysis, manuscript drafting. Christina M. Ray: Manuscript editing for intellectual content. Katherine A. Buhler: Supervision, data analysis, manuscript editing for intellectual content. Satchel Krawchuk: Data collection, manuscript editing for intellectual content. Humberto Jijon: Manuscript editing for intellectual content. Cathy Lu: Manuscript editing for intellectual content. Cynthia H. Seow: Manuscript editing for intellectual content. Kerri L. Novak: Manuscript editing for intellectual content. Joelle St-Pierre: Manuscript editing for intellectual content. Siddharth Singh: Manuscript editing for intellectual content. Remo Panaccione: Manuscript editing for intellectual content. Gilaad G. Kaplan: Data analysis, manuscript editing for intellectual content. Christopher Ma: Supervision, data analysis, manuscript drafting, manuscript editing for intellectual content.

Conflicts of Interest:
The authors disclose no conflicts.

Funding:
The authors report no funding.

Ethical Statement:
This is an analysis of anonymized, publicly available data; ethics approval was waived.

Data Transparency Statement:
Data used in this analysis are publicly available from the Healthcare Cost and Utilization Project National Inpatient Sample.

Reporting Guidelines:
Reporting Guidelines were not applicable for this article type.

Gastro Hep Advances, Volume *5*

Supplemental information

Significant Increase in *Clostridioides difficile* Mortality During the COVID-19 Pandemic: A Nationwide Study

Jonathan Besney, Christina M. Ray, Katherine A. Buhler, Satchel Krawchuk, Humberto Jijon, Cathy Lu, Cynthia H. Seow, Kerri L. Novak, Joelle St-Pierre, Siddharth Singh, Remo Panaccione, Gilaad G. Kaplan, and Christopher Ma

Supplemental Table 1. ICD-10 codes used for analysis

Diagnosis	ICD-10 codes
C. difficile infection	A04.7 enterocolitis due to *C. difficile*<br>B96.8 other specified bacterial agents as the cause of diarrhea<br>R19.58 other fecal abnormalities
C. difficile carrier status (exclusionary)	Z22.1 carrier of other intestinal infectious diseases<br>Z22 carrier of other specified bacterial diseases<br>Z86.19 personal history of other infectious and parasitic diseases<br>Z87.1 personal history of diseases of the digestive system
